# Research progress in the role and mechanism of LPCAT3 in metabolic related diseases and cancer

**DOI:** 10.7150/jca.71619

**Published:** 2022-05-01

**Authors:** Gaoxuan Shao, Yufan Qian, Lu Lu, Ying Liu, Tao Wu, Guang Ji, Hanchen Xu

**Affiliations:** 1 Institute of Digestive Diseases, Longhua Hospital, Shanghai University of Traditional Chinese Medicine - Shanghai 200032, China.; 2 Institute of Interdisciplinary Integrative Medicine Research, Shanghai University of Traditional Chinese Medicine, Shanghai, China.

**Keywords:** Lysophosphatidylcholine acyltransferase 3, Phosphatidylcholine, Atherosclerosis, Inflammation, Nonalcoholic steatohepatitis, Cancer

## Abstract

Lysophosphatidylcholine acyltransferases (LPCATs) are among the lysophopholipid acyltransferases (LPLATs) that specifically regulate the abundance of different phosphatidylcholine (PC) species in a variety of cell and tissue types, thereby playing an important role in lipid metabolism and homeostasis. Lysophosphatidylcholine acyltransferase 3 (LPCAT3, MBOAT5) is a member of the LPCAT family that primarily regulates the levels of arachidonic PC species. LPCAT3 is regulated by the liver X receptor, which plays an important role in lipoprotein production in the liver and small intestine. Increasing lines of research have demonstrated that LPCAT3 plays important roles in the occurrence and development of many diseases, such as atherosclerosis, intestinal tumors, and nonalcoholic steatohepatitis (NASH). The development of many diseases has been linked to the proinflammatory effects of LPCAT3. This review focuses on the current knowledge of LPCAT3, including its function and mechanism in different diseases. We aim to provide a comprehensive and in-depth understanding of LPCAT3 and to provide new ideas for the treatment of some diseases.

## Introduction

Phospholipids are the major components of biological membranes, and the major structural phospholipids of mammalian membranes are glycerophospholipids, including phosphatidylcholine (PC), phosphatidylethanolamine (PE), phosphatidylserine (PS), phosphatidylinositol (PI) and phosphatidic acid (PA). Of these phospholipids, PC is the most abundant phospholipid in mammalian cell membranes and subcellular organelles, accounting for approximately 40-50% of total phospholipids 1. In mammals, fatty acids are first activated to acyl coenzyme A. Through the use of acyl coenzyme A, PC is synthesized via the *de novo* synthesis pathway, also known as the Kennedy pathway 2,3.

However, the acyl groups in glycerophospholipids show a high degree of diversity and are distributed in an asymmetric manner that cannot be fully explained by the *de novo* synthesis pathway 4, 5. Saturated and monounsaturated fatty acids are usually esterified at the sn-1 position of glycerophospholipids, while polyunsaturated acyl groups are located at the sn-2 position. Lands described the rapid conversion of the sn-2 acyl fraction of glycerophospholipids as a remodeling pathway (Lands' cycle) 6. Thus, after *de novo* synthesis of phospholipids, the fatty acyl chains in the sn-2 position of phospholipids are hydrolyzed by phospholipase A2 (PLA2) to produce 1-acyl lysophospholipids, which react with lysophospholipid acyltransferase (LPLAT) to incorporate another fatty acid into the sn-2 position and form a new phospholipid species.

Lysophosphatidylcholine acyltransferases (LPCATs) are LPLAT enzymes that specifically regulate the abundance of different PC species in a variety of cell and tissue types and thus play an important role in lipid metabolism and homeostasis. LPCATs belong to two distinct families based on their amino acid sequences. LPCAT1 and LPCAT2 are members of the acylglycerol phosphate acyltransferase family, which contains four conserved structural domains, called LPA acyltransferase motifs 1-4 7, 8, and endoplasmic reticulum (ER) localization sequences 9. On the other hand, LPCAT3 and LPCAT4 (also known as MBOAT5 and MBOAT2, respectively) belong to the membrane-bound O-acyltransferase (MBOAT) family. They contain the MBOAT motif but lack the LPA acyltransferase motif 10,11. Additionally, LPCAT3 and LPCAT4 are ER membrane proteins. Increasingly, LPCAT3 has been found to play an important role in the development and progression of diseases such as atherosclerosis, NASH, intestinal tumors, diabetes, and skeletal muscle myopathy and is likely to be a potential therapeutic target for these diseases in the future. Therefore, in this review, we focus specifically on LPCAT3 and provide a macroscopic perspective on the role and mechanisms that LPCAT3 plays in the development of different diseases in a comprehensive manner.

## Structure and function of LPCAT3

LPCAT3, also known as MBOAT5, belongs to the MBOAT family. LPCAT3 contains the MBOAT motif but lacks the LPA acyltransferase motif 10,11. As an ER membrane protein, LPCAT3 also contains a C-terminal ER retention signal, KKXX 12 and has 487 amino acids, a molecular weight of 56 kDa, and seven algorithm-identified 13 transmembrane domains. LPCAT shows different tissue distributions, enzymatic activities and substrate preferences. LPCAT3 is very widely expressed and is abundantly present in the testes, kidneys and metabolic tissues, including liver, intestine and adipose tissues 10,14,15. In addition to its main lysoPC acyltransferase activity, LPCAT3 is active against lysoPE and lysoPS as substrates 10. A recent study found that LPCAT3 is the primary source of bulk lyso-PS acyltransferase activity in the mouse brain. More importantly, LPCAT3 and ABHD12, a gene encoding an integral membrane lysophosphatidylserine (lysoPS) lipase, synergistically regulate lysoPS and C20:4 PS content in the CNS (central nervous system), and lysoPS lipids may be bioactive metabolites in neuropathology associated with PHARC (polyneuropathy, hearing loss, cerebellar ataxia, retinitis pigmentosa, early-onset cataracts) 16. Most importantly, each LPCAT exhibits a different acyl coenzyme preference. LPCAT3 prefers polyunsaturated fatty acyl-CoA (18:2-acyl-CoA or 20:4-acyl-CoA) as a substrate 10,15. LPCAT3 plays a major role in regulating the levels of polyunsaturated PLs in the cell membrane system, especially arachidonic PC species 17-19.

LPCAT3 is responsible for most LPCAT activity in certain cell types 10,14, and reduced LPCAT3 activity disrupts Golgi structures and limits plasma membrane extension and cell proliferation. LPCAT3 is regulated by the liver X receptor 20, which plays an important role in lipoprotein production in the liver and small intestine 21,22. In addition to LXR, LPCAT3 is also regulated by nuclear receptors PPARs (peroxisome proliferator-activated receptors), mainly PPARα and PPARδ 14,23, which play important roles in fatty acid (FAs) and glucose metabolism 24-26. In particular, activation of PPARδ prevents endoplasmic reticulum stress, inflammation and insulin resistance in different metabolic tissues, including skeletal muscle and liver. Intestinal LPCAT3 activity is required for the gut-brain feedback loop that integrates lipid absorption with food intake 21. LPCAT3 deficiency reduces lipid absorption and thus circulating atherogenic lipoproteins 27-29. LPCAT3 deficiency increases skeletal muscle insulin sensitivity through the regulation of plasma membrane PC components 30 and impairs circulating atherogenic lipoproteins through the activation of the Wnt/β-linked protein pathway, impairing preadipocyte adipogenesis 31.

## Role of LPCAT3 in different diseases

### Atherosclerosis and LPCAT3

Arachidonic acid (AA) is a precursor to important lipid mediators such as leukotrienes and prostaglandins that induces inflammation and is associated with the progression of atherosclerosis 32. LPC is also considered a proinflammatory molecule 33 and has been found to enhance inflammation by increasing free fatty acid concentrations that activate PLA2 34; therefore, controlling LPC levels as well as AA levels may inhibit the development of atherosclerosis. LPC levels were increased in atherosclerotic tissues of people experiencing chronic inflammation 35-37, and as the pathological grading of atherosclerosis worsened, the accumulation of LPC in the tissues increased and the corresponding arachidonic acid-PC levels decreased. In addition, we detected higher levels of LPCAT3 expression in the arachidonic acid-PC accumulation zone than in the LPC accumulation zone at each pathological stage 35. LPCAT3 is a major regulator of free AA levels, which are a key factor associated with the development of atherosclerosis 38,39. It has been found that changes in arachidonic acid-PC and LPCAT3 levels in atherosclerotic tissues may lead to alterations in free AA levels. In addition, there is abundant LPCAT3 expression in vascular smooth muscle cells, which plays an important role in the progression of atherosclerosis 35. Therefore, the modulation of arachidonic acid-PC accumulation and LPCAT3 expression in vascular smooth muscle cells may be a potential means to inhibit the progression of atherosclerosis, which remains to be further demonstrated.

Atherosclerosis has been shown to be closely associated with inflammation and to exist in close association with macrophages, with macrophage-derived foam cells in the vessel wall producing many proinflammatory chemokines and cytokines 40, thereby promoting atherogenesis. The treatment of macrophages with lipopolysaccharide (LPS) or peptidoglycan promoted the assembly of Toll-like receptor (TLR) complexes in lipid rafts 41-43, which play an important role in TLR4-mediated signaling 42, 44. We found that reduced levels of macrophage plasma membrane sphingolipids could effectively prevent inflammatory responses by reducing TLR4 expression 45-47, thereby reducing atherosclerosis 45,46,48. It has also been reported that cellular lipids are important regulators of C-SRC activation and can alter the recruitment of C-SRC to the plasma membrane 49. Many studies have also shown that C-SRC plays a key role in macrophage-mediated inflammatory responses 50. A recent report showed that C-SRC phosphorylation (activation)-mediated NFκB activation and elevated TNFα can be involved in macrophage activation and inflammatory responses 51. More importantly, LPCAT3 deficiency significantly increased phosphorylated C-SRC in macrophage lipid rafts 35. At the same time, an LPCAT3 deficiency-mediated reduction in macrophage plasma membrane polyunsaturated PC levels induces TLR4 expression in lipid rafts, which in turn induces MAP kinase and NFκB activation and promotes inflammatory cytokine production 35. Thus, LPCAT3 can exert proinflammatory effects through both the activation of C-SRC and a reduction in TLR4 expression. This would seem to suggest that macrophage LPCAT3 deficiency may exacerbate atherosclerosis by promoting an inflammatory response. However, recent studies have found that bone marrow cell-specific LPCAT3 deficiency does not significantly change atherogenesis 35, whereas hematopoietic cell-specific LPCAT3 deficiency promotes atherosclerosis, and the transplantation of hematopoietic cells from constitutive LPCAT3-deficient mice in LDLR-/- mice leads to increased atherosclerotic lesions 52.

In summary, in macrophages, LPCAT3 exerts a proinflammatory effect by regulating the expression of C-SRC and TLR4, thereby promoting the further development of atherosclerosis. At the same time, LPCAT3 can also regulate two inflammatory proinflammatory factors, LPC and AA, in vascular smooth muscle, thereby exacerbating atherosclerosis.

### Skeletal muscle myopathy and LPCAT3

Muscle weakness is one of the main features of skeletal muscle myopathy, and obesity leads to a reduction in certain lipid species in the muscle, leading to the development of muscle weakness and severe skeletal myopathy. Through a comprehensive lipid profile analysis, we found that skeletal muscle lysoPC was positively correlated with maximal force production capacity. Interestingly, previous studies have found that obesity leads to a reduction in muscle lysoPC 30. Additionally, in skeletal muscle, LysoPC is mainly regulated by LPCAT3, which is acylated to produce PC, and consistent with this finding, lysoPC is reduced when skeletal muscle LPCAT3 is overexpressed 53. Thus, we suggest that LPCAT3 may be closely associated with the development of skeletal muscle myopathy through the regulation of lysoPC expression.

A recent study has tested these hypotheses. This study found slight contractile dysfunction in skeletal muscle after the overexpression of LPCAT3 in the skeletal muscle of mice. In contrast, in HFD-fed skeletal-muscle-overexpressing LPCAT3 mice, significantly reduced lysoPC levels and severe myopathy were observed 54. Thus, LPCAT3 overexpression induces skeletal muscle myopathy, which is further exacerbated by obesity and is most likely mediated through lysoPC. This provides a new therapeutic target for the treatment of obesity-induced skeletal myopathy.

### NASH and LPCAT3

Recent studies have revealed certain links between LPCAT3 and the development and progression of NASH. First, LPCAT3 expression is regulated by LXR. Lpcat3 expression is increased by LXR activation, which promotes the entry of unsaturated fatty acids into PLs and reduces ER membrane saturation, thereby reducing ER stress and liver inflammation. In contrast, acute knockdown of hepatic LPCAT3 exacerbates ER stress and liver inflammation 15. Moreover, ER stress activates sterol regulatory element binding protein (SREBP) through the ATF6, PERK and IRE1 signaling pathways, promoting lipid accumulation in the liver 55. Studies have demonstrated that a disruption of IRE1A or the inhibition of IRE1A in hepatocytes then reduces the release of inflammatory extracellular vesicles (EVs) and decreases liver injury and inflammation in NASH mice 56. Therefore, we predicted that in NASH patients, hepatic LPCAT3 expression should be reduced, thereby exacerbating ER and liver inflammation. However, a recent study found that the mRNA and protein expression levels of LXRα and LPCAT3 were significantly elevated in the liver of a NASH mouse model. In the study, NASH was induced in mice through an HFD. Surprisingly, the mRNA levels of the ER-mediated signaling molecules perk, atf4, atf6, and ire1α, which are associated with inflammation, were significantly higher in the liver tissues of mice in the HFD group than in the NC group 57.

Another study also measured LPCAT3 expression in a NASH mouse model and found opposite results. The NASH mice in this study were induced by an HFDS (high fat diet + sucrose). NASH mice had significantly lower expression of LPCAT3 mRNA and significantly higher LPC content than control mice. When shRNA-mediated knockdown of Lpcat3 in Huh-7 cells (shLpcat3 cells) was treated with palmitic acid (PA), the intracellular LPC concentration and cell death were significantly higher than those in wild-type Huh-7 cells. In contrast, intracellular LPC and palmitic-acid-induced cell death were significantly lower in LPCAT3-overexpressing Huh-7 cells than in wild-type cells 58. PA is a saturated fatty acid (FA) that induces apoptosis in hepatocytes, and LPC is also known to be a causative agent of apoptosis in liver-injured hepatocytes. Previous studies have found that serum free fatty acids (FFAs) are higher in NASH patients than in healthy subjects 59. Therefore, toxic lipids, such as saturated FFAs, are a suspected cause of NASH. It was also found that LPCAT3 overexpression decreased PA-induced expression of CHOP (a transcription factor that acts as a stress marker for the endoplasmic reticulum) and attenuated PA-induced cell death 59. These results suggest that LPCAT3 may be a therapeutic target for NASH by reducing hepatocyte death.

Although we cannot yet determine why two experiments measuring LPCAT3 expression in NASH mice yielded opposite results, it is certain that LPCAT3 expression is closely linked to the progression of NASH. The exact relationship and the corresponding mechanisms need to be further investigated.

### Diabetes and LPCAT3

One enzyme that plays a major role in diet-induced cardiometabolic disease is group 1B phospholipase A2 (PLA2G1B), which catalyzes fat digestion in the intestinal lumen after feeding 60. In rodent models, chemical inhibition and genetic inactivation of PLA2G1B are protective against the obesity and hyperglycemia induced by chronic feeding of a high-fat/high-carbohydrate diet 61,62.

When liver LPCAT3-overexpressing mice and control mice were fed a mixed liposaccharide diet separately, the control mice showed significant glucose tolerance abnormalities, whereas LPCAT3-overexpressing mice showed improved postprandial hyperglycemia and glucose tolerance 63, consistent with the phenotype of pla2g1b-/- mice. The improved postprandial glucose tolerance in pla2g1b-/- mice was due to elevated fatty acid β-oxidation in the livers of these animals 64, 65. Interestingly, the same conclusion was reached in LPCAT3-overexpressing mice. Mitochondrial fatty acid β-oxidation was significantly higher in LPCAT3-overexpressing mice than in controls, and this was achieved by reducing intracellular LPC levels 62, which has also previously been shown to cause mitochondrial dysfunction 64. Therefore, we suggest that LPCAT3 overexpression increased the utilization of phospholipid synthesis by lysophospholipids, thereby attenuating the inhibition of fatty acid oxidation by lysophospholipids and improving glucose tolerance.

Surprisingly, however, the overexpression of LPCAT3 in skeletal muscle led to the opposite result. Skeletal muscle is the site of the greatest glucose handling in humans 66,67. Insulin resistance in skeletal muscle is a necessary precursor to type 2 diabetes 68 and can be triggered by abnormal lipid metabolism 69-71. The overexpression of skeletal muscle-specific LPCAT3 is sufficient to enhance HFD-feeding-induced glucose intolerance in mice. Conversely, the inhibition of LPCAT3 enhanced insulin signaling at the IR level and improved skeletal muscle insulin sensitivity 30. Similarly, *in vitro* experiments yielded similar findings. Human skeletal muscle cells (HSKMCs) were collected from insulin-sensitive and lean (ln) or insulin-resistant and obese (ob) subjects, and human primary muscle cells from ob subjects were more insulin-resistant than those from ln controls 30. LPCAT3 deletion was followed by enhanced insulin-stimulated glycogen synthesis, suggesting that this intervention increased skeletal muscle insulin sensitivity *in vitro*. LPCAT3 knockdown also enhanced insulin signaling in HSKMCs from obese subjects 30.

As previously described, we learned that hepatic LPCAT3 overexpression improves glucose tolerance by reducing LPC levels and thereby enhancing mitochondrial fatty acid β-oxidation. However, LPCAT3 overexpression in skeletal muscle exacerbates glucose intolerance, and the inhibition of its expression enhances insulin signaling and improves insulin sensitivity, thereby improving insulin resistance. Modern medical research has found that patients with low glucose tolerance can develop diabetes and that 10%-50% of patients with low glucose tolerance become clinically diabetic after 10 years of follow-up. At the same time, it has been found that insulin resistance is prevalent in type II diabetes, accounting for almost 90% of cases, and may be an important initiating factor in the development and progression of type II diabetes. However, we are currently unsure whether there is a direct link between LPCAT3 and the development of diabetes, and this needs to be further investigated.

### Tumors of the intestine and LPCAT3

The few intestinal stem cells (ISCs) in the intestinal crypts continuously divide to give rise to daughter stem cells and highly proliferative progenitor cells called transitional amplification (TA) cells 72. A proper balance between ISC self-renewal and differentiation is essential for maintaining the integrity of the intestinal epithelium and maintaining homeostasis 73. Homeostasis dysregulation in the intestine is known to lead to serious intestinal diseases, including cancer. Existing studies have found that LPCAT3 is closely associated with intestinal stem cell proliferation and the development of intestinal tumors. It has been found that intestinal LPCAT3 deficiency leads to the overproliferation of intestinal crypts and the overproliferation of ISCs and progenitor cells and induces intestinal tumorigenesis 74,75. It is now mainly believed that the above results occur due to the increased cholesterol synthesis following intestinal LPCAT3 deficiency, which drives ISC and progenitor cell proliferation and ISC self-renewal and promotes tumor formation.

In epidemiological studies, cholesterol intake has been associated with an increased risk of gastrointestinal cancers 76. In addition, we know that excess exogenous (dietary) cholesterol stimulates ISC proliferation in WT mice, a change that is thought to be induced by the activation of PPAR and ultimately enhances the tumorigenic potential of ISCs and allows nonstem cell progenitors to differentiate to form living tumors 77. Similarly, endogenous cholesterol synthesis was found to similarly stimulate ISCs, as learned from the analysis of intestine-specific SREBF2 transgenic mice, which had enhanced cholesterol biosynthesis and increased cellular cholesterol levels and whose ISC overproliferation could be clearly observed 75. More importantly, after intestinal LPCAT3 deficiency, free cholesterol levels in crypts were increased by 25% compared to controls. In the APC^min^ mouse model, cholesterol biosynthesis was enhanced following intestinal LPCAT3 deficiency, stimulating ISC proliferation and significantly contributing to intestinal tumorigenesis 74,75. Conversely, the proliferation of ISCs in intestinal LPCAT3 mice was significantly reduced when LSS, a cholesterol synthesis inhibitor, was administered 74.

Thus, we suggest that intestinal LPCAT3 deficiency is likely due to the promotion of cholesterol production, which induces excessive ISC proliferation and further promotes intestinal tumor formation.

### Other cancers and LPCAT3

Within the tumour microenvironment (TME), tumor-associated macrophages (TAMs) represent one of the most abundant immune cell types and are characterised by heterogeneity and plasticity, resulting in populations ranging from anti-tumour to pro-tumour tams1. Emerging evidence suggests that dysregulated lipid metabolism not only enhances the metastatic and invasive behaviour of cancer cells 78, 79 but also contributes to the production of lipid-rich TMEs, which impede host anti-tumour immunity and maintain the survival of suppressive cells 80-82. Endoplasmic reticulum stress, in particular IRE1-mediated production of X-box binding protein 1 (XBP1) and the spliced form of C/EBP homologous protein (CHOP), has recently been found to be associated with the promotion of T cell dysfunction 83, 84, impairment of dendritic cell antigen presentation capacity 85 and an immunosuppressive phenotype of myeloid-derived suppressor cells (MDSC) 86. Recent studies have found that TAMs display high lipid content and ER stress responses, while ER stress and dysregulated lipid metabolism may be coordinated to tailor the pro-tumorigenic profile of TAMs. Tumour cells can preferentially increase lipid content and tumourigenic polarisation of macrophages via the IRE1-XBP1 pathway in an IL-4/IL-13 non-dependent manner. Expression of XBP1 drives TAM in a pro-tumourigenic direction, thus providing a survival advantage to the tumour. Genetic ablation of Xbp1 in mouse bone marrow cells inhibited tumour growth and was accompanied by a significant loss of TME macrophages 87. IRE1 contains a transmembrane structural domain that senses lipid imbalance and induces its dimerisation and activation 88 and increased cholesterol accumulation may realign the lipid composition of ER membranes, resulting in a lower ratio of phosphatidylcholine to phosphatidylethanolamine (PC/PE ratio). This reduces ER membrane fluidity as cholesterol sensing mechanisms are disturbed 89. LPCAT3 has been shown to limit lipid overload-induced ER stress in hepatocytes 15. LPCAT3 overexpression decreased the PC/PE ratio and unsaturated PC abundance on ER membranes in YUMM1.7 tumor cell-derived conditioned medium (CM) treated bone marrow (BM)-derived macrophages (BMDM). More importantly, LXR-mediated induction of macrophage LPCAT3 can be used to reawaken the anti-tumour response 87.

Ferroptosis is a newly discovered form of non-apoptotic cell death driven by the catastrophic accumulation of iron-dependent lipid reactive oxygen species (ROS) and is morphologically, biochemically and genetically distinct from traditional cell death such as apoptosis, necrosis and autophagy 90. Ferroptosis can be induced by iron accumulation, glutathione (GSH) depletion, glutathione peroxidase 4 (GPX4) inactivation, and is characterised by lipid peroxidation products and toxic reactive ROS generated by iron metabolism 91,92. Emerging research suggests that ferroptosis is associated with a wide range of human diseases, including cancer, degenerative diseases, carcinogenesis, stroke, cerebral haemorrhage, trauma, brain injury, ischaemia-reperfusion injury and renal degeneration 91. Targeting ferroptosis -related genes (FRGs) to trigger ferroptosis has attracted considerable attention as a new therapeutic approach for cancer diagnosis and treatment 93.Wang et al. identified a 3-FRG (HIC1, LPCAT3, DUOX1) profile to predict the prognosis of ovarian cancer (OC) patients. Patients in the high-risk group had a shorter median overall survival (OS) and a poorer prognosis 94. Of the three genetic markers, HIC1, LPCAT3 and DUOX1, in their prognostic model, HIC1 was the most studied gene associated with cellular immune function. However, for the role of LPCAT3 in this, it is only a prognostic marker and the exact role and mechanism of LPCAT3 in ferroptosis is not known. Similarly, the role of LPCAT3 in ferroptosis in brain metastases from breast cancer (BCBM) is still in its early stages and is currently similarly stuck in marker status. Although Stockwell et al. found that LPCAT3 knockdown would inhibit ferroptosis 91, we still need to develop *in vitro* and *in vivo* experimental models to assess the role of LPCAT3 in BCBM.

With the above discussion, we find that the promise of LPCAT3's potential therapeutic role in cancer is enormous. It can exert anti-tumour effects by regulating ER stress. Meanwhile, its role in ferroptosis in addition to that of a marker, the mechanism by which LPCAT3 exerts specific anti-cancer effects needs to be further investigated.

## Conclusion and Perspectives

LPCAT3, as the main LPCAT in the liver, intestine and adipose tissue, especially the liver and intestine 10,14,15, is now increasingly being studied, with a focus on its role in liver- and intestine-related diseases or the mechanisms by which it is involved. It has been shown that Lpcat3 is closely associated with the formation and development of atherosclerosis, but because its selective expression in different cells or tissues either promotes or inhibits atherosclerosis, it is important to conclude what role LPCAT3 expression in different cells and tissues plays in atherosclerosis. In the context of intestinal tumorigenesis, current studies suggest that low expression of LPCAT3 in the intestine is likely to induce ISC overproliferation and further promote intestinal tumor formation by promoting cholesterol production 72. Little research has been done on the relationship between LPCAT3 and NASH, but studies have identified a link between the two, and it is likely that LPCAT3 promotes the development and progression of NASH through ER stress 15. It has also been found that LPCAT3 overexpression or knockdown in skeletal muscle promotes the development of skeletal myopathy and insulin resistance, respectively 30,54.

LPCAT3 is currently of most interest for its role in inhibiting tumourigenesis and progression, but little research has been done in this area. Although LPCAT3 has now been shown to inhibit ferroptosis, it is not known whether it can subsequently inhibit tumor progression. The research is still limited to its role as a marker for breast and ovarian cancer, but more research is needed to determine what role LPCAT3 plays in cancer or what its mechanism of action is. However, we are pleased that the latest research has demonstrated that LPCAT3 may exert anti-tumor effects through the IRE1-XBP1 pathway of the ERs. Even more surprisingly, that LXR, the upstream regulator of LPCAT3, may act through its agonist to increase LPCAT3 expression. Therefore, our future studies could be further investigated into the relationship between LPCAT3 and ERs and the tumorigenesis and progression associated with ERs. In particular, we know that ERs is closely related to the development of inflammation, which can further lead to the development of cancer, such as NASH-driven HCC (hepatocellular carcinoma). More importantly, we have known that LPCAT3 is the predominant LPCAT in the liver and regulates hepatic ERs contingency. Therefore, it is reasonable to wonder whether LPCAT3 can inhibit the NASH to HCC transition.

**Figure 1:**
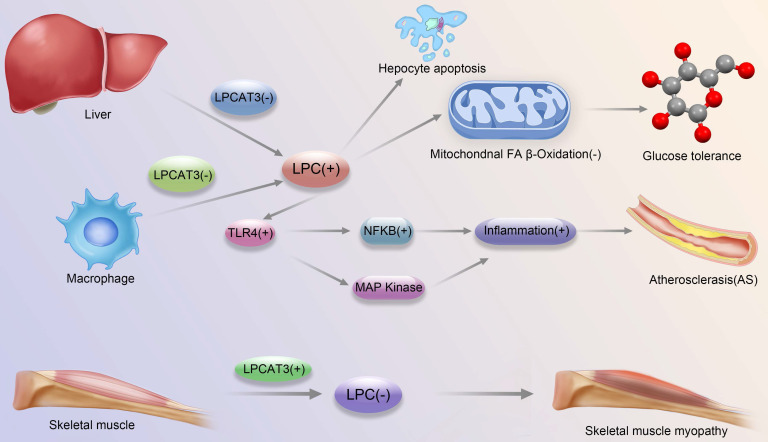
** Development and progression of major diseases associated with LPCAT3-regulated LPC-related pathways.** Liver LPCAT3 deficiency leads to increased LPC synthesis, which leads to: 1. apoptosis of hepatocytes 2. diminished mitochondrial fatty acid beta oxidation, which in turn affects glucose tolerance; Macrophage LPCAT3 deficiency leads to increased LPC synthesis, which in turn enhances TLR4 expression, which regulates enhanced expression of MAPK and NFkB pathways, respectively, which in turn promotes inflammation, leading to the promotion of atherosclerosis development; LPCAT3 overexpression in skeletal muscle cells leads to decreased LPC synthesis and contributes to the development of skeletal muscle myopathy.

This review focuses on the structural and functional characteristics of LPCAT3, especially the progress of research on the potential mechanisms involved in different diseases, such as atherosclerosis, intestinal tumors, NASH, skeletal myopathy and insulin resistance, and identifies some patterns of LPCAT3 in the development of these diseases (Fig. 1). At the same time, due to the wide distribution of LPCAT3, it is reasonable to assume that it may also act in other diseases associated with the tissues or organs in which it is abundantly present, and this needs to be further investigated and explored. More research is also needed to understand the mechanisms by which LPCAT3 acts in the above known diseases and to uncover the molecular pathways that act in these diseases. In conclusion, LPCAT3 is likely to be an effective target for the treatment and prevention of the various diseases mentioned above. Therefore, LPCAT3 targeted therapy will be a promising therapeutic strategy, but the current research results on LPCAT3-targeted therapy are minimal, and this will be a popular direction for future research, which is also important for the discovery of new therapeutic ideas and approaches.

## Abbreviations

LPCAT: lysophosphatidylcholine acyltransferases
LPLAT: lysophopholipid acyltransferase
LPCAT3: lysophosphatidylcholine acyltransferase 3
PC: phosphatidylcholine
NASH: non-alcoholic fatty liver hepatitis
PE: phosphatidylethanolamine
PS: phosphatidylserine
PI: phosphatidylinositol
PA: phosphatidic acid
PLA2: phospholipases A2
ER: endoplasmic reticulum
MBOAT: membrane-bound O-acyltransferase
AA: arachidonic acid
LPC: lysophosphatidylcholine
LPS: lipopolysaccharide
TLR: Toll-like receptor
C-SRC: cell sarcoma
NFκB: nuclear factor-k-gene binding
TNF: tumor necrosis factor
MAPK: mitogen-activated protein kinase
LDLR: Low-Density Lipoprotein Receptor
ISC: intestinal stem cell
TA: transitional amplification
PPAR: peroxisome proliferators-activated receptor
SREBF2: sterol regulatory element-binding transcription factor 2
APC: adenomatous polyposis coli
LSS: lanosterol synthase
LXR: liver X receptor
PL: phospholipid
SREBP: sterol regulatory element binding protein
ATF6: Activating transcription factor 6
PERK: protein kinase R-like endoplasmic reticulum kinase
IRE1: inositol-requiring enzyme 1
EVS: extracellular vesicles
FFA: free fatty acid
CHOP: C/EBP homologous protein
PLA2G1B: group 1B phospholipase A2
HSKMC: human skeletal muscle cell
ERs: endoplasmic reticulum stress
CNS: central nervous system
PPARs: peroxisome proliferator-activated receptors
TME: tumour microenvironment
TAM: tumor-associated macrophage
XBP1: X-box binding protein 1
MDSC: myeloid-derived suppressor cells
CM: conditioned medium
BMDM: bone marrow derived macrophages
ROS: reactive oxygen species
GPX4: glutathione peroxidase 4
FRG: ferroptosis -related gene
OC: ovarian cancer
OS: overall survival
BCBM: brain metastases from breast cancer
HCC: hepatocellular carcinoma
